# Single-Cell Transcriptomes Combining with Consecutive Genomics Reveal Clonal Evolution and Gene Regulatory Networks in Relapsed and Refractory Multiple Myeloma

**DOI:** 10.3389/fcell.2021.794144

**Published:** 2022-01-05

**Authors:** Jiadai Xu, Yue Wang, Zheng Wei, Jingli Zhuang, Jing Li, Yifeng Sun, Liang Ren, Yawen Wang, Panpan Li, Shiyang Gu, Yian Zhang, Jifeng Jiang, Chen Chen, Yu Zhang, Peng Liu

**Affiliations:** ^1^ Department of Hematology, Zhongshan Hospital, Fudan University, Shanghai, China; ^2^ Department of Cardiovascular Surgery, Renji Hospital, School of Medicine, Shanghai Jiaotong University, Shanghai, China

**Keywords:** multiple myeloma, clonal evolution, relapsed, refractory, heterogeneity

## Abstract

This study attempted to investigate how clonal structure evolves, along with potential regulatory networks, as a result of multiline therapies in relapsed/refractory multiple myeloma (RRMM). Eight whole exome sequencing (WES) and one single cell RNA sequencing (scRNA-seq) were performed in order to assess dynamic genomic changes in temporal consecutive samples of one RRMM patient from the time of diagnosis to death (about 37 months). The 63-year-old female patient who suffered from MM (P1) had disease progression (PD) nine times from July 2017 [newly diagnosed (ND)] to Aug 2020 (death), and the force to drive branching-pattern evolution of malignant PCs was found to be sustained. The mutant-allele tumor heterogeneity (MATH) and tumor mutation burden (TMB) initially exhibited a downward trend, which was then upward throughout the course of the disease. Various somatic single nucleotide variants (SNVs) that had disappeared after the previous treatment were observed to reappear in later stages. Chromosomal instability (CIN) and homologous recombination deficiency (HRD) scores were observed to be increased during periods of all progression, especially in the period of extramedullary plasmacytoma. Finally, in combination with WES and scRNA-seq of P1-PD9 (the nineth PD), the intro-heterogeneity and gene regulatory networks of MM cells were deciphered. As verified by the overall survival of MM patients in the MMRF CoMMpass and GSE24080 datasets, RUNX3 was identified as a potential driver for RRMM.

## Introduction

Multiple myeloma (MM) is a plasma cell malignancy that is characterized by highly intra-clonal heterogeneity. Despite emerging novel therapeutic strategies, response to treatment and final outcomes continue to vary in MM patients, and the disease is considered to be mostly incurable. During MM progression, therapeutic pressure may bestow a selective advantage on sub-clonal expansion through which the fittest subclone will dominate. In turn, the presence of sub-clonal mutations may affect the subsequent efficacy of the targeted therapy, resulting in a vicious cycle. The development of anti-MM therapy requires an understanding of driver genetic alterations, gene regulatory networks as well as an appreciation of the evolutionary processes of malignant plasma cells (PCs).

Recently, large-scale genomic sequencing studies have provided deep insights into the genetic landscape of MM (Robiou du Pont et al., 2017; Walker et al., 2018), which may provide potential targeted candidates for personalized therapy. However, most of these studies obtained only a single, or at most two, samples from each individual patient. Little is known about the temporal clonal evolutionary processes in refractory or multiple relapsed MM. In 2012, three landmark studies provided insight into the intra-clonal heterogeneity early at the diagnosis and different stages of MM after relapse (Egan et al., 2012; Keats et al., 2012; Walker et al., 2012), suggesting a Darwinian model of tumor evolution in MM.

In this study, whole exome sequencing (WES) and single cell RNA-sequencing (scRNA-seq) are simultaneously applied in order to determine dynamic genomic changes among eight temporal consecutive samples of one relapsed and refractory MM (RRMM) patient from the time of diagnosis to death (about 37 months). By searchingdatasets from the Multiple Myeloma Research Foundation (MMRF) CoMMpass (Clinical Outcomes in MM to Personal Assessment of Genetic Profile) study (*n* = 766) and Gene Expression Omnibus (accession GSE24080, *n* = 559), RUNX3, a member of runt-related transcription factor family (referred to as RUNXs), is shown to serve as a potential driver for RRMM or secondary plasma cell leukemia (PCL). Accordingly, from genomics to single cell transcriptomes, the manner in which the clonal structure and regulatory networks evolved under the pressure of multiline therapies was investigated.

## Materials and Methods

### Patients and Samples

A 63-year-old female patient with MM (P1) suffered progressive disease (PD) nine times from July 12, 2017 (new diagnosis (ND)) to August 8, 2020 (death) at Zhongshan Hospital, Fudan University. Since she also suffered from coronary atherosclerotic heart disease and undergone twice percutaneous coronary intervention in the period from ND to PD2, she missed benefiting from the best opportunity for receiving autologous hematopoietic stem cells. Eight temporal consecutive samples (ND, PD1, PD2, PD3, PD4, PD6, PD8, and PD9) were collected. The samples from ND to PD6 were CD138 positive PCs from bone marrow, while PD8 sample were tissues from extramedullary plasmacytoma. In the end-stage of MM (PD9), malignant PCs appeared in her peripheral blood (PB), the proportion of which reached 10%. The patient refused to undergo bone marrow aspiration again. Therefore, for PD9, CD138 positive PCs from PB were used for WES, PB mononuclear cells (PBMC) were used for scRNA-seq.

The diagnosis for MM, International Staging System stage (ISS), and Durie-Salmon stage (DS) were determined in accordance with the criteria of International Myeloma Working Group (IMWG), 2018 (Kumar and Rajkumar, 2018). The definition of progressive disease (PD) adhered to the IMWG consensus criteria for response in 2016 (Kumar et al., 2016). Time to progression (TTP) was calculated from the initiation of therapy to progression. Electronic records of this RRMM patient were reviewed. Written informed consent was provided by the patient according to the Declaration of Helsinki. This study was approved by the ethics committee of Fudan University, Zhongshan Hospital (B2017-031R).

### Collection of Mononuclear Cells and CD138+/CD138- Cell Sorting

Collecting mononuclear cells from bone marrow (BM) aspirate and peripheral blood (PB), along with sorting CD138+/CD138- plasma cells from BM mononuclear cells (BMMC), were performed as previously described (Xu et al., 2020).

### Deoxyribonucleic Acid Extraction, Library Construction and Whole Exome Sequencing Basic Data Analysis

Genomic DNA were extracted from the sorted CD138 + plasma cells and matched PB mononuclear cells (PBMC) by utilizing the QIAamp DNA Mini kit (250) (51,306, QIAGEN). DNA was subsequently quantified using the Qubit 3.0 (Invitrogen) and Nanodrop spectrophotometer (Thermo Fisher Scientific), while integrity was assessed using 1% agarose electrophoresis. Genomic libraries were then captured using the Agilent SureSelect Human All Exon V6 kit (Agilent Technologies, United States). Approximately 2–3 μg genomic DNA was sheared to 150–220 bp small fragments using a sonicator (Covaris, Inc., Woburn, MA). DNA was purified and treated with reagents supplied with the kit according to the given protocol. Adapters from Agilent were ligated onto the polished ends, and the libraries were amplified using polymerase chain reaction. The amplified libraries were then hybridized with the Agilent SureSelect Human All Exon V6 (Müeller-Pillasch et al., 1997) probes. The DNA fragments bound with the probes were washed and eluted with the buffer provided in the kit. These libraries were sequenced on the Illumina sequencing platform (HiSeq X-10, Illumina, Inc., San Diego, CA), after which 150 bp paired-end reads were generated.

The raw data (FASTQ format) were pre-processed with fastp (version: 0.19.5) (Chen et al., 2018). Reads containing less than 70% bases with average quality value below 20 (Q20) were filtered out using the NGSQC toolkit (version 2.3.2). Bases with a quality below Q20 were trimmed from the 3’ end. Reads with ambiguous bases or those shorter than 75 bp were also removed. High quality and clean reads were aligned to the reference human genome (GRCh37/hg19) using the Burrows-Wheeler aligner (BWA) (version 0.7.12) (Li and Durbin, 2009). Duplicate reads were removed using Picard (version 4.1.0.0). The mapped reads were sorted and indexed using the Sequence Alignment Map tool (SAMtools, version 1.4) (Li et al., 2009). The Genome Analysis Toolkit (GATK, version 4.1.0.0) (McKenna et al., 2010) was used for the recalibration of base quality score and realignment of single nucleotide polymorphisms (SNPs) and short insertion/deletions (INDELs). The final BAM files were used as input files for variant calling. Variants were annotated using ANNOVAR (Wang et al., 2010). The somatic mutations including somatic single nucleotide variants (SNVs) and somatic INDELs were screened out by comparing the variants between CD138^+^ plasma cells and the matched PBMC using MuTect2 (version 1.1.7) (Cibulskis et al., 2013). Identification of copy number alterations (CNA) were called using the Control-FREEC software (Boeva et al., 2012). The whole exome sequencing was conducted by OE Biotech Co., Ltd (Shanghai, China).

### Clonal Evolution Analysis

All somatic mutations of the eight consecutive samples at different stages (ND-PD1-PD2-PD3-PD4-PD6-PD8-PD9) were merged. Nonsynonymous somatic mutations and INDELs that changed the protein amino acid sequence were then filtered out. The sites with maf >0.01 in the population database of 1000 g, exac, gnomad, esp6500, as well as sites with a depth of less than 30, were then removed. Pyclone software was used to analyze the clone type of each sample. Based on the results of Pyclone, ClonEvol was used to analyze the evolution of clones between samples. According to the ClonEvol findings, the fishplot R package was used to draw a fish pattern (Roth et al., 2014).

### Screening of Known Driver Genes

In order to identify the mutated driver genes, all somatic variations in each sample were then compared with the known driver genes. The sources of the driver genes for comparison were: 1) CGC513: driver gene listed in Cancer Gene Census list; 2) Bert Vogelstein125 (Vogelstein et al., 2013): 125 mut-driver genes in Bert Vogelstein’s paper; and 3) SMG127: a significantly mutated gene found via TCGA pan-cancer data. The potential driver genes in these MM samples were screened out.

### DNA Ploidy Profiling

Hypodiploidy or whole chromosome deletion is defined by a total of less than 45 whole chromosomes. However, hyper-diploidy is defined by chromosome amplification in the form of trisomies or tetrasomies of odd number chromosomes (48–74 chromosomes). Estimation of ploidy was analyzed using the Sequenza2 software (Favero et al., 2015).

### Tumor Mutation Burden, Homologous Recombination Deficiency Scores and Mutant-Allele Tumor Heterogeneity

TMB = Number of nonsynonymous somatic mutations in the area of coding sequence (CDS)/Length of CDS. HRD scores were calculated as previously described using the scarHRD R package (Davies et al., 2017). MATH = 100 × median absolute deviation (MAD)/median (Mayakonda et al., 2018).

### Chromium 10× Single-Cell 3' mRNA Sequencing and Data Processing

The main steps of scRNA-seq for PD9 were cell preparation, cDNA synthesis, library construction, and sequencing. This protocol refers specifically to the CG00052_SingleCell3_ReagentKitv2UserGuide_RevD, which is downloadable from the 10× Genomics website. The three PBMC samples of the healthy people were taken from official Chromium 10× (PBMC1 https://cg.10xgenomics.com/samples/cell-exp/3.0.0/pbmc_10k_v3/pbmc_10k_v3_fastqs.tar;PBMC2 https://cg.10xgenomics.com/samples/cell-exp/4.0.0/Parent_NGSC3_DI_PBMC/Parent_NGSC3_DI_PBMC_fastqs.tar; PBMC3 https://cg.10xgenomics.com/samples/cell-exp/4.0.0/SC3_v3_NextGem_DI_PBMC_10K/SC3_v3_NextGem_DI_PBMC_10K_fastqs.tar). The Cell Ranger software pipeline (version 3.1.0) provided by 10 × Genomics was used to demultiplex cellular barcodes, map reads to the genome and transcriptome using the STAR aligner, and down-sample reads as required to generate normalized aggregate data across samples, thereby producing a matrix of gene counts versus cells. The Seurat R package (version 3.1.1) was used to process the unique molecular identifier (UMI) count matrix, remove low quality cells and likely multiplet captures, obtain the normalized count and gene expression, perform graph-based clustering and identify marker genes of each cluster. Cells were visualized using a 2-dimensional t-distributed stochastic neighbor embedding (t-SNE) algorithm. Differentially expressed genes (DEGs) were identified using the FindMarkers function. *p* value <0.05 and |log2foldchange| > 0.58 were set as the thresholds for significantly differential expression. Cell trajectory was carried out by Monocle. Single-cell regulatory network inference, and clustering (SCENIC) analysis was performed in order to infer the regulon activity score (Hänzelmann et al., 2013; Trapnell et al., 2014; Haghverdi et al., 2016).

## Results

### Interpretation of Therapy-Induced Evolution of MM Cells Through Analyzing Eight Temporal Consecutive WES Data

**FIGURE 1:**
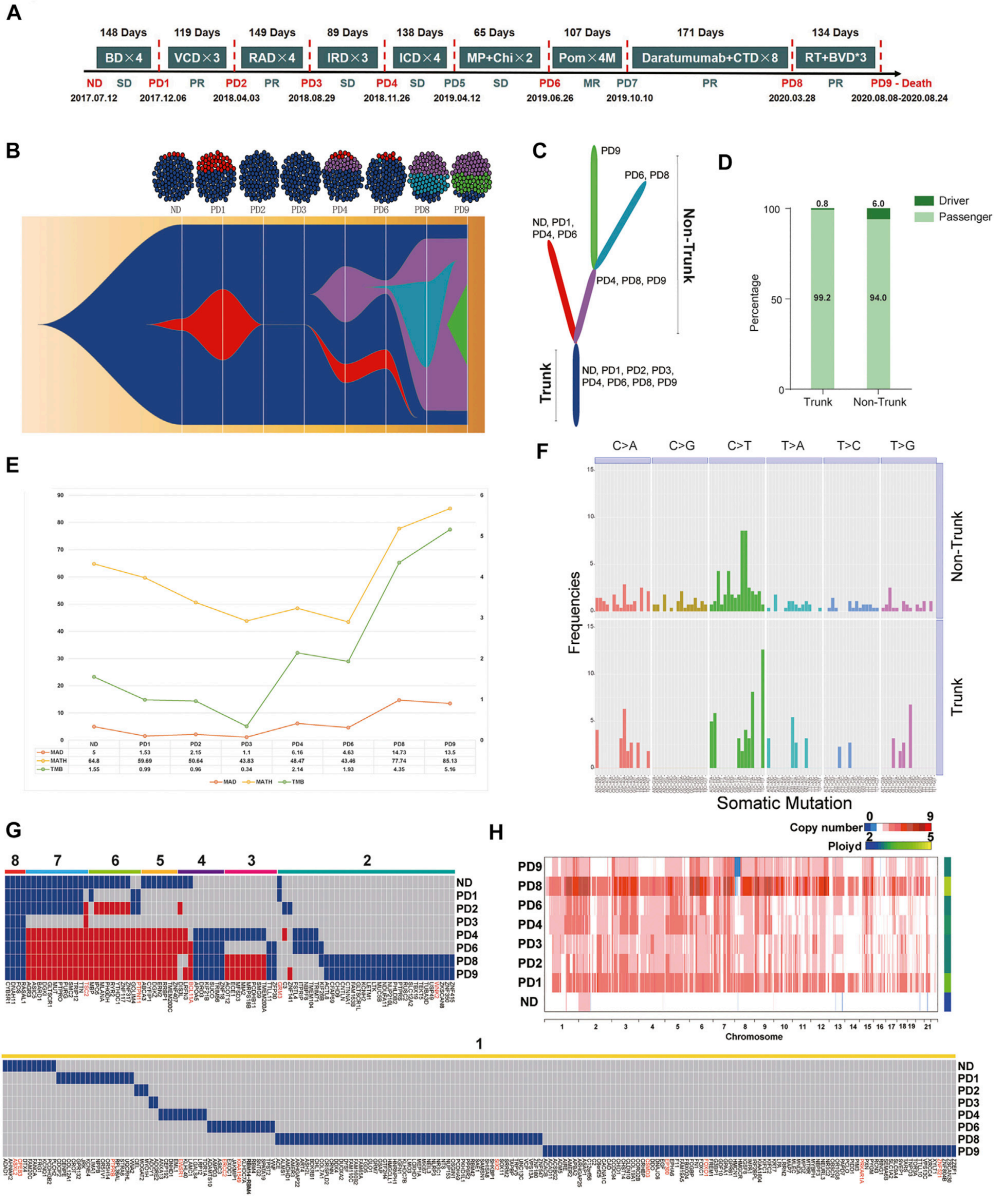
**(A)** Treatment timeline for P1: After diagnosis with active MM (2017.7.12), P1 received Bortezomib/Dexamethasone (BD) as the primary therapy. After four cycles of BD, P1 suffered the first PD (PD1, day 1–148, TTP = 148 days). The second line therapy were Bortezomib/Cyclophosphamide/Dexamethasone (VCD). After three cycles of VCD, P1 suffered the second PD (PD2, day 148–266, TTP = 119 days). Lenalidomide/Adriamycin/Dexamethasone (RAD) were selected as the third line therapy regimen. The third PD (PD3, day 266–414, TTP = 149 days) occurred after three cycles of RAD. After three cycles of the fourth-line therapy, Ixazomib/Lenalidomide/Dexamethasone (IRD), P1 suffered the fourth PD (PD4, day 414–503, TTP = 89 days). Ixazomib/Cyclophosphamide/Dexamethasone (ICD) were the fifth-line therapy regimen. After four cycles of ICD, P1 had a clinical relapse (PD5, day 503–640, TTP = 138 days). The therapy further changed to Melphalan/Prednisone/Chidamide (MP + Chi). However, P1 had another PD (PD6, day 640–704, TTP = 65 days). The patient received Pomadomide (Pom) as the seventh-line therapy until the seventh PD (PD7, day 704–811, TTP = 107 days). The eighth-line therapy was Daratumumab and Cyclophosphamide, Thalidomide and Dexamethasone (CTD) which sustained 171 days until extramedullary plasmacytoma occurred on May 28, 2020 (PD8, day 811–982, TTP = 171 days). Finally, P1 received radiotherapy and Bendamustine/Bortezomib/Dexamethasone (BVD) for three cycles. In the end-stage of disease, malignant PCs appeared in her peripheral blood (PB), the proportion of which reached 10%. She passed away on Aug 24th, 2020. **(B)** Fish model showing heterogeneity of malignant PCs from P1 was analyzed by Pyclone and ClonEvol. After four times internal balances between red cluster and dark blue cluster (ND-PD3), a new clonal cluster (purple) was acquired under the pressure of multiple-line therapy interventions on PD4. Malignant PCs of PD9 consisted of 1) the always-existing dark-blue trunk sub-group, 2) the purple non-trunk sub-group evolved from PD4, and 3) the newly emerging green non-trunk sub-group. **(C)** The branching-pattern phylogenetic relationships in all malignant PCs from P1. **(D)** These driver genes were scattered along the phylogenetic tree, surprisingly, the number of driver genes in the non-trunk cluster (6.0%) was higher than the trunk cluster (0.8%). **(E)** From the first diagnosis to the nineth relapse, MATH of each sample was 64.80, 59.65, 50.64, 43.83, 48.47, 43.46, 77.74, and 85.13. TMB of each sample was 1.55, 0.99, 0.96, 0.34, 2.14, 1.93, 4,35 and 5.16, respectively. **(F)** Among all mutations, C > T transitions were the predominant change. In addition, T [C > T]T (12.61%) and T [C > T]A (8.11%) transitions dominated the trunk mutations, while G [C > T]G and G [C > T]C transitions (8.57 and 8.57%) dominated the non-trunk mutations. **(G)** The temporal distribution of depth >50, copy number >0 and nonsynonymous SNVs detected by WES in a heat map, with dark blue or red indicating the presence of a mutation and gray indicating the absence of a mutation. The eight color bars above the heat map indicate classification of these SNVs according to the total number of occurrences in the samples. For the gene names, red indicates that the mutation maybe a known driver gene, and black indicates a passenger gene. **(H)** Heterogeneity at the copy number (CN) level was analyzed. The ploidy of ND-PD9 samples were 2.029, 3.532, 2.768, 2.753, 2.873, 2.817, 4.556, and 2.774, respectively.

Treatment timeline for P1 is shown in Figure 1A. WES was conducted with a mean coverage depth of 167× (range: 126-220X) for CD138 + plasma cells and 173× for PBMCs (control), consistent with the recommendations for WES. The vast majority of genomic sequences (99.78–99.97%) were mapped to the hg19 (GRCh37) reference genome. The detailed clinical data of baseline, as well as each progression of P1, are given in Supplementary Table S1. The total number of different types of somatic single nucleotide variants (SNVs) and short insertion/deletions (INDELs), summary of quality control and mapping results in the eight consecutive samples are summarized in Supplementary Figure S1, Supplementary Table S2 and Supplementary Table S3, respectively.

In order to investigate the changes in the clonal architecture and decipher the clonal evolution of MM cells in light of different treatments, Pyclone and ClonEvol were used. Heterogeneity (Figure 1B) and the branching-pattern phylogenetic relationships in these malignant PCs were then detected (Figure 1C). Notably, after conducting internal balances between red cluster and dark blue cluster (ND-PD3) four times, a new clonal cluster (purple) was acquired under the pressure of multiple-line therapy interventions on PD4. Branch lengths of the phylogenetic tree were found to be proportional to the number of nonsynonymous mutations separating the branching points. The trunk (the dark blue cluster), which were the most persistent clones, existed throughout the entire course and formed the mutational profile of the founder population. The non-trunk cluster, namely, the red cluster, purple cluster, light blue cluster and green cluster, were present in some stages.

According to the known driver genes from annotations of three pan-cancer databases, including CGC513, BV125 and SMG127, a total of 18 driver variants were identified (Supplementary Table S4). These driver genes were found to be scattered along the phylogenetic tree. Surprisingly, the number of driver genes in the non-trunk cluster (6.0%) was observed to be higher than that of the trunk cluster (0.8%) (Figure 1D). This indicated that the force to drive branching-pattern evolution of malignant PCs was sustained. In view of the heterogeneity and complex regulating network in MM progression, trunk and non-trunk genes were enriched into multiple pathways and functions using the Gene Ontology (GO) databases (Supplementary Figure S2).

From first diagnosis to the nineth relapse, the MATH of each sample was determined to be 64.80, 59.65, 50.64, 43.83, 48.47, 43.46, 77.74, and 85.13. Meanwhile, the TMB of each sample was 1.55, 0.99, 0.96, 0.34, 2.14, 1.93, 4,35, and 5.16, respectively. The trend of MATH and TMB presented an initial gradual downward trend followed by an upward trend throughout the course of the disease (Figure 1E). PD3 showed the lowest MATH and TMB in the eight continues samples. Moreover, only the dark blue cluster (Trunk) was detected in PD3, which was actually an imbalanced status of the bulk malignant PCs. In conjunction with the clinical data presented in Figure 1A, this status actually led to the shortest TTP as well as a strong and rapid counterattack of the red and purple clusters (PD4). Accordingly, we attempted to understand this phenomenon from an evolutionary perspective; administration according to a fixed and linear protocol is a selective perturbation that may lead to the emergence of drug-resistant subclones. Dynamic imbalances may not be conducive to stabilizing malignant MM cells. Adaptive therapy based on evolutionary law may provide a potential strategy for RRMM treatment to attain a fixed tumor population and prolong PFS rather than reducing malignant cells entirely.

The trinucleotide mutational spectrum of trunk (up) and non-trunk (down) mutations based on the phylogenetic tree is shown in Figure 1F. Among all mutations, C > T transitions were found to be the predominant change. In addition, T [C > T]T (12.61%) and T [C > T]A (8.11%) transitions dominated the trunk mutations, while G [C > T]G and G [C > T]C transitions (8.57 and 8.57%) dominated the non-trunk mutations.

During therapy, most MM patients initially go into remission, however, drug-resistant mutations may later cause disease progression. The mutational spectrum was further analyzed based on the timeline of mutation acquisition (Figure 1G). This figure illustrates the temporal distribution of depth >50, copy number >0 and nonsynonymous SNVs detected by WES in a heat map, with dark blue or red indicating the presence of a mutation and gray indicating the absence of a mutation. The eight color bars above the heat map indicate classification of these SNVs according to the total number of occurrences in the samples. In terms of gene names, red indicated that the mutation may be a known driver gene, while black indicated a passenger gene. Here, only four identical somatic passenger mutations (FOS, RASAL1, CYB5R1, and DNAH11) were found to be ubiquitously detectable in all samples. No known-driver gene was shared between the eight specimens. By observing the genomics of P1, some SNVs that had disappeared after the previous treatment were observed to reappear in later stages (red blocks). Mutations were further classified into susceptible SNVs and resistant SNVs for each treatment course. GO and Kyoto Encyclopedia of Genes and Genomes (KEGG) enrichment analyses were conducted, as shown in Supplementary Figure S3 and Supplementary Figure S4. Based on personal genomics, a potential treatment strategy—recycle therapy may be considered.

The heterogeneity at the copy number (CN) level was then analyzed (Figure 1H). The ploidy of ND-PD9 samples were found to be 2.029, 3.532, 2.768, 2.753, 2.873, 2.817, 4.556, and 2.774, respectively. Remarkably, compared to the ND sample, the PD samples were observed to possess higher-ploidy karyotypes, indicating that chromosomal instability (CIN) increased during the period of progression. Among the PD samples, PD8 (extramedullary plasmacytoma) had the highest ploidy after the eighth-line therapy, which combined Daratumumab with Cyclophosphamide, Thalidomide and Dexamethasone (CTD).

HRD scores of each sample are summarized in Table 1, for which the HRD-sum scores [Loss of Heterozygosity (LDH) + Number of Telomeric Allelic Imbalances (TAI) + Large Scale Transitions (LST)] of each sample were found to be 5, 63, 44, 50, 64, 52, 67, and 60. In MM, extramedullary progression is always associated with treatment resistance as well as a high mortality rate. The changing trend of HRD scores were observed to be consistent with ploidy.

**TABLE 1:** HRD-scores of the P1 patient.

Samples ID	LOH*	TAI*	LST*	HRD-sum*
ND	0	3	2	5
PD1	0	39	24	63
PD2	0	36	8	44
PD3	0	32	18	50
PD4	0	37	27	64
PD6	0	31	21	52
PD8	0	39	28	67
PD9	0	34	26	60

Abbreviations: LOH: Loss of Heterozygosity; TAI: Number of Telomeric Allelic Imbalances; LST: Large Scale Transitions; HRD-sum: Heterozygosity scar.

### Combination With Genomics and Single Cell Transcriptomes Deciphered the Heterogeneity and Distinct Evolutional Subpopulations in MM Cells

Next, the single-cell transcriptome data of PD9, from peripheral blood of P1 (about 10% PCs), were analyzed. After conducting quality control, 553 356 446 sequence reads and 39 525 reads per cell for an estimated 14 000 cells were obtained, with 87.3% confidently mapped to the human reference transcriptome GRCh38–3.0.0. On average, 937 genes and 2,369 unique molecular identifiers (UMIs) per cell were detected.

**FIGURE 2:**
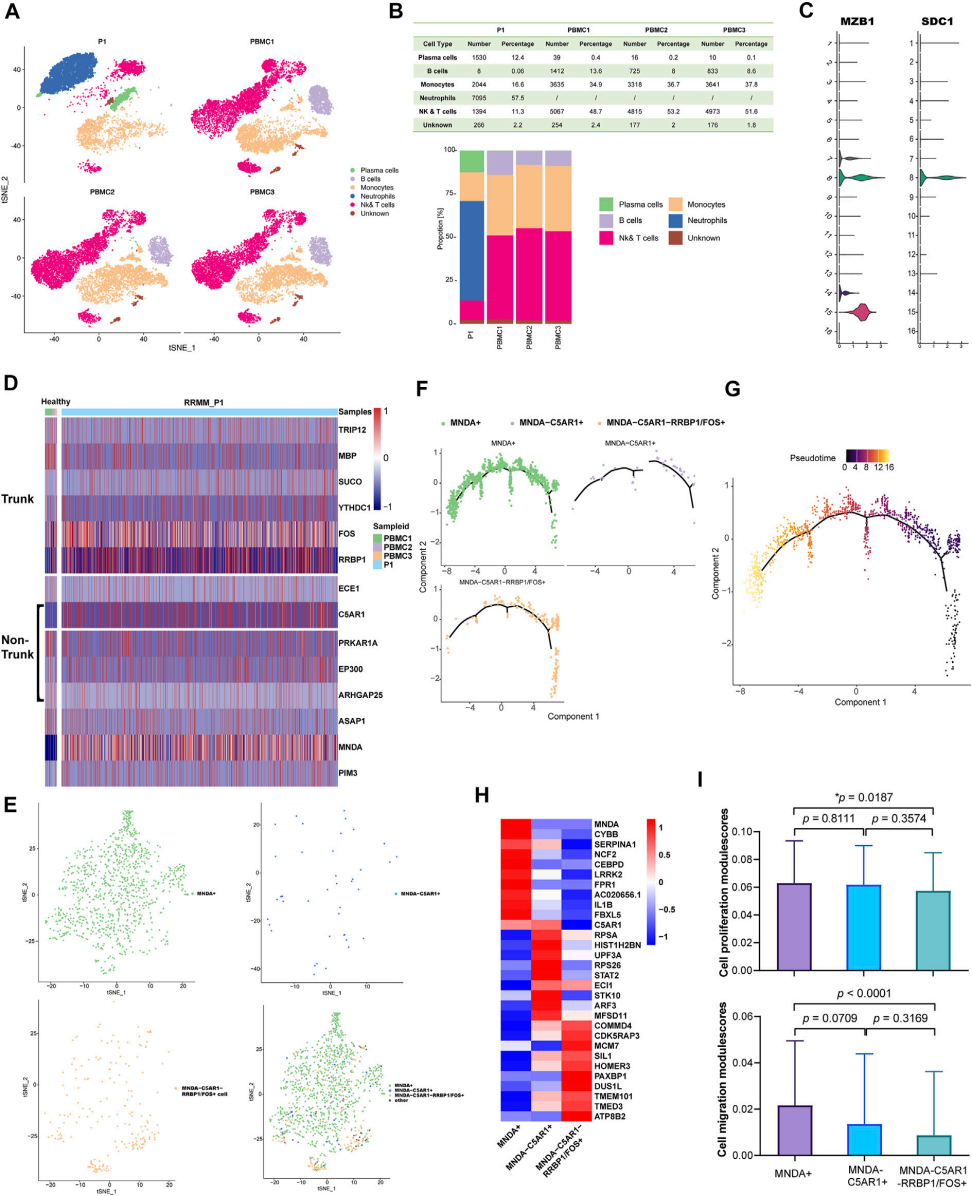
**(A)** Comparing with the data of three healthy PBMC controls from the 10× platform database described above, cell types were identified in P1_PD9 and were visualized by t-SNE (16 clusters in total). **(B)** Stacked bar plots show that compared with normal specimens, plasma cells significantly increased and B cells significantly decreased in the P1 sample. **(C)** According to gene markers, including SDC1 (CD138) and MZB1, cluster 8 was finally determined to be the malignant PCs. **(D)** According to the WES results of evolutionary tree from P1 in Figure 1B and the mutational profiling of trunk and non-trunk, the mRNA expression levels of these mutant genes between PCs from normal control (PBMC1, 2, 3) and malignant PCs from P1 were compared and showed in the heatmap. **(E)** Malignant PCs in P1 was further grouped as three sub-clusters: 1) PCs expressed higher level of MNDA (MNDA+, the green non-trunk sub-group in WES results); 2) PCs expressed higher level of C5AR1 but no MNDA (C5AR1+ MNDA-, the purple non-trunk sub-group in WES results); and 3) PCs expressed higher level of FOS or RRBP1 but no MNDA and C5AR1 (MNDA-C5AR1-RRBP1/FOS+, the dark-blue trunk sub-group in WES results). **(F)** Minimum spanning tree (MST) of malignant PCs performed by pseudo-time analysis also revealed that the MNDA + non-trunk sub-group evolved from MNDA-C5AR1-RRBP1/FOS + trunk sub-group (from right to left). **(G)** The pseudo-time of malignant PCs. **(H)** The different expression gene profiles of the three sub-clusters. **(I)** Cell proliferation and migration modulescores among the three sub-group.

In order to explore the cellular composition of PBMC from P1_PD9, a comparison was made with the data of three healthy PBMC controls from the 10 × platform database described in supplementary methods, while unsupervised clustering was applied to distinguish the cell types. Finally, PCs, B cells, monocytes, neutrophils, nature killer cells (NK) and T cells were categorized and visualized using t-distributed stochastic neighbor embedding (t-SNE) (Figure 2A). Stacked bar plots (Figure 2B) demonstrated that, compared to normal specimens, PCs were significantly increased in the P1 sample. Identification of PCs subpopulations (cluster 8) was then performed according to gene markers, including SDC1 (CD138) and MZB1 (Figure 2C).

According to the above WES results of evolutionary tree from P1 (Figures 1B,C), malignant PCs of PD9 consisted of: 1) the always-existing dark-blue trunk sub-group; 2) the purple non-trunk sub-group evolved from PD4; and 3) the newly emerging green non-trunk sub-group. In view of the mutational profiling of the above sub-groups, the expression levels of these mutant genes between PCs from normal control (PBMC1, 2, 3) and P1 were then compared (Figure 2D). Genes that were not expressed in the displayed cells were filtered out and not shown on the heatmap. Based on the results of the heatmap, PCs from P1 was further grouped as three sub-clusters: 1) PCs expressed higher level of MNDA (MNDA+, the green non-trunk sub-group); 2) PCs expressed higher level of C5AR1 but no MNDA (C5AR1+ MNDA-, the purple non-trunk sub-group); and 3) PCs expressed higher level of FOS or RRBP1 but no MNDA and C5AR1 (MNDA-C5AR1-RRBP1/FOS+, the dark-blue trunk sub-group) (Figure 2E). Minimum spanning tree (MST) of malignant PCs performed by pseudo-time analysis verified that the MNDA + sub-cluster evolved from MNDA-C5AR1-RRBP1/FOS + sub-cluster (Figures 2F,G). The different expression profiles of the three sub-clusters are illustrated in the corresponding heatmap (Figure 2H), reflecting dynamic gene expression profiles during the malignant evolution of MM cells. The AddModuleScore tool from Seurat was then utilized in order to calculate the module scores for cell proliferation and cell migration expression in the three sub-clusters, respectively. Compared to the trunk cluster, the MNDA + cluster exhibited significantly higher scores in cell proliferation (*p* = 0.0187) and cell migration (*p* < 0.0001) (Figure 2I). Together, the corresponding data highlighted the heterogeneity and 180 distinct evolutional subpopulations among MM cells.

### Gene Regulatory Networks Revealed by scRNA-seq Identify RUNX3 Gene as a Potential Driver for RRMM

**FIGURE 3:**
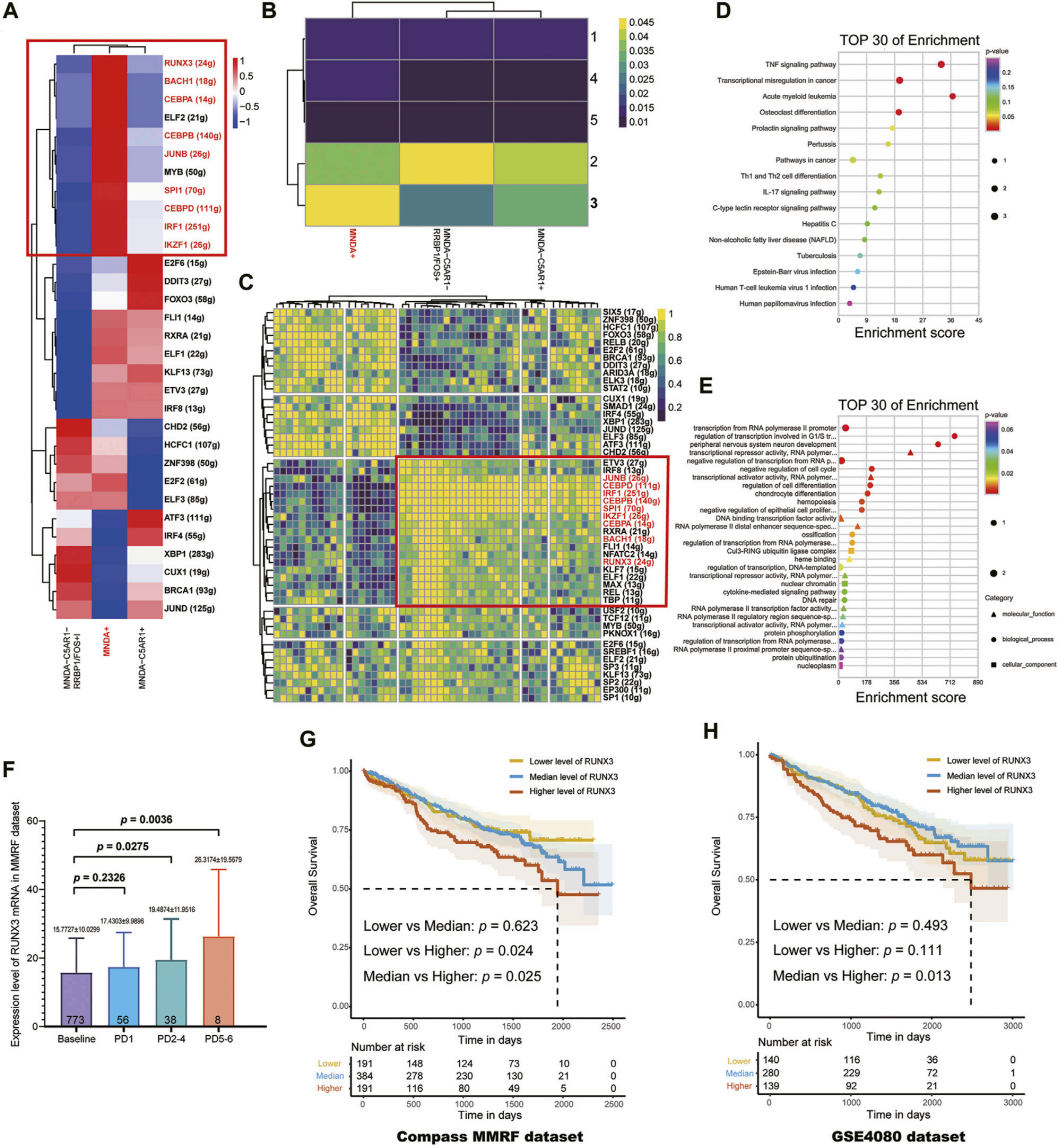
**(A)** The regulon activity score (RAS) for MNDA + sub-cluster, C5AR1+ MNDA- sub-cluster and MNDA-C5AR1-RRBP1/FOS + sub-cluster, respectively. **(B)** Five regulon modules were identified for the three sub-groups by the Connection Specificity Index. **(C)** Inter-gene expression correlations and specific involved genes in each module. **(D)** Enrichment analysis using KEGG of 9 TFs. **(E)** Enrichment analysis using GO of 9 TFs. **(F)** Progressive increase of RUNX3 mRNA expression level with times of PD (Baseline vs. PD 2–4: *p* = 0.0275, Baseline vs. PD 5-6: *p* = 0.0036) using the MMRF-CoMMpass datasets. **(G)** RUNX3 mRNA expression level was found to be the only poor prognostic factor among the 9 TFs (OS, low vs. high: *p* = 0.024, median vs. high: *p* = 0.025) in 766 newly diagnosed MM. **(H)** The survival effect of RUNX3 gene was validated in GSE24080 dataset (median vs. high: *p* = 0.013).

Transcription factors (TFs) and their targeted genes comprise a complex gene network regulation, referred to as a regulon, that could determine cell functional identity. Single-cell regulatory network inference and clustering (SCENIC) analysis was performed to infer the regulon activity score (RAS) for the MNDA+, C5AR1+ MNDA- and MNDA-C5AR1-RRBP1/FOS + sub-clusters, respectively (Figure 3A). Five regulon modules were then identified for the three sub-groups according to the Connection Specificity Index (CSI), indicatinga significant correlation between different regulons with minimization of the effects of non-specific crosstalk (Figure 3B). Inter-gene expression correlations as well as the specifically involved genes in each module are shown in Figure 3C.

In terms of the most malignant sub-cluster, namely the MNDA + sub-cluster, the intersection of TFs marked with red boxes in Figures 3A,B were determined by incorporating the results of regulation activity and specific modules. Enrichment analysis using KEGG (Figure 3D) and GO (Figure 3E) of the 9 TFs, including JUNB, CEBPD, IRF1, CEBPB, SPL1, IKZF1, CEBPA, BACH1, RUNX3, suggested that the TNF signaling pathway, transcriptional mis-regulation in cancer, regulation of transcription involved in G1/S transition of mitotic cell cycle may serve vital roles.

Next, using the MMRF-CoMMpass datasets, a progressive increase of RUNX3 mRNA expression level with times of PD were found (Baseline vs. PD 2-4: *p* = 0.0275, Baseline vs. PD 5-6: *p* = 0.0036) (Figure 3F). Moreover, the overall survival (OS) of the 766 newly diagnosed MM (NDMM) were calculated *via* Kaplan-Meier survival analysis in order to estimate the effect of the above 9 TFs. Accordingly, by univariate survival analysis (Kaplan-Meier analysis, log-rank test), the RUNX3 mRNA expression level was found to be the only poor prognostic factor among the 9 TFs (low vs. high: *p* = 0.024, median vs. high: *p* = 0.025) (Figure 3G). Furthermore, multivariate survival analysis by Cox regression, including age, Eastern Cooperative Oncology Group performance status (ECOG) and the Revised International Staging System (R-ISS), identified the RUNX3 gene as an independent prognostic factor for OS (Table 2, *p* = 0.027). The survival effect of the RUNX3 gene was then validated in the GSE24080 dataset (Kaplan-Meier analysis, log-rank test, median vs high: *p* = 0.013) (Figure 3H). While for the multivariate survival analysis, including age, cytogenetic abnormalities, albumin and beta-2 micro-globulin, RUNX3 showed a trend to be an independent prognositic factor for OS (Table 3, *p* = 0.070). The corresponding findings suggest that the RUNX3 gene may be a potential therapeutic target for the treatment of MM.

**TABLE 2:** Multivariate analysis of OS in MMRF compass dataset.

Variable	HR	95% CI	*p*-value
RUNX3	1.028	1.003–1.053	0.027
Age	1.047	1.028–1.067	0.000
ECOG	1.364	1.102–1.689	0.004
R_ISS	2.191	1.571–3.057	0.000

Abbreviations: CI, confidence interval; HR, hazard ratio; ECOG: Eastern Cooperative Oncology Group performance status; R-ISS: the Revised International Staging System.

**TABLE 3:** Multivariate analysis of OS in GSE24080 dataset.

Variable	HR	95% CI	*p*-value
Age	1.015	0.998–1.031	0.083
RUNX3	1.254	0.982–1.601	0.070
ALB	0.698	0.558–0.873	0.002
β2M	1.067	1.047–1.088	0.000
Cyto abnor	2.096	1.548–2.838	0.000

Abbreviations: CI, confidence interval; HR, hazard ratio; Cyto abnor: the detection of cytogenetic abnormalities; ALB: Albumin; β2M: Beta-2 microglobulin.

## Discussion

In this study, temporal consecutive genetic analysis provided evidence of heterogeneity in MM and unmasked the evolutionary trajectories of PCs derived by multi-line therapies. Based on somatic mutations, the evolution trajectory was constructed to reflect the development of this RRMM patient, showing that the dominant pattern was branch evolution. Final scRNA-seq data provided new evidence and insights into deciphering the heterogeneity and evolution in RRMM, which are pivotal in dissecting MM-related mechanisms in detail. We hypothesized that MM clonal evolution and progression underlie Darwinian selection, which are mediated by tumor-intrinsic characteristics as well as extrinsic pressure.

In the past decades, the survival of most MM patients has improved significantly due to the development of autologous stem cell transplantation (ASCT) and novel agents, including proteasome inhibitors, immunomodulatory drugs, and monoclonal antibodies (Kumar et al., 2008). Despite its overall improvement, a group of high-risk patients with clinically aggressive behaviors (PFS less than 18 months, OS less than 1.5–3 years) continues to suffer. Current therapies rarely consider molecular information to personalized MM therapy. In order to continually improve MM patients’ outcomes, information regarding the genomic abnormalities leading to these heterogenous outcomes should be incorporated into personal clinical care (Pawlyn and Davies, 2019). Recently, high-throughput sequencing has brought about personalized treatment according to the specific genetic composition and molecular phenotype of patients. Notably, in this study, a majority of known MM driver genes previously reported did not actually exist in this individual’s MM genome sequences (Robiou du Pont et al., 2017), suggesting that MM is a highly heterogeneous malignant disease, and personal omics is crucial in clinical management. This is conducive in developing individualized targeted medication and identifying potential personal therapeutic targets. In this case, mutations may be classified into susceptible SNVs and resistant SNVs for each of P1’s treatment courses. Disappearance of susceptible somatic mutations and emergence of resistant somatic mutations may provide guidance for personalized-therapy. Accordingly, agents effective against these recurring mutations during the earlier stages may be re-administrated. The alternate use of agents not only conforms to the theory of adaptive therapy, but also provides a therapeutic solution for multi-line recurrence MM patients.

In regard to MATH and TME, dynamic imbalance was found to not be conducive to stabilizing malignant MM cells. In 2009, Gatenby et al. (2009) first put forward the adaptive therapy theory and presented powerful mathematical models to represent their findings. They pointed out that resistant populations that are present before therapy would rapidly grow with treatments designed to kill the majority of malignant cells, which is a result of removal of the inhibitory effect that the sensitive population has on the resistant population, in conjunction with the disturbed balance between both populations. Adaptive therapy takes advantage of the inhibitory effect of the sensitive population on the resistant population, leading to the slow growth of entire cancer cells. A study on the spatial and temporal clonal evolution of intrahepatic cholangiocarcinoma also provided a theoretical basis for adaptive therapy (Dong et al., 2018). Recently, An et al. (2020) reported that persistent cytogenetic abnormalities were detected in residual PCs in the majority of MM patients, which were even present in the minimal residual disease (MRD) negative cohort. These findings may further provide evidence for the potential effectiveness of adaptive therapy in MM treatment.

Moreover, understanding the trajectories of the temporal consecutive changes of PCs may serve as a powerful tool in estimating risk of progression and could bring about profound implications in clinical management. According to Charlotte Pawlyn and Gareth J Morgan (Pawlyn and Morgan, 2017), subsequent driver lesions often occur in a sub-clonal PCs in order to facilitate MM progression. In the patient presented in this study, driver genes were observed to be scattered along the phylogenetic tree, suggesting that the force to drive branching-pattern evolution of malignant PCs was sustained. In addition, a total of four identical passenger mutations were present in all samples: FOS, RASAL1, CYB5R1, and DNAH11. Accordingly, we hypothesized that the four mutations may be closely associated with the proliferation of MM cells. FOS, Fos proto-oncogene or AP-1 transcription factor subunit, belongs to the Fos gene family and encodes leucine zipper proteins that dimerize with proteins of the JUN family (Cannarile et al., 2019). FOS proteins have established roles as regulators of cell proliferation, differentiation, and apoptosis. According to Youg Raj Thaker et al., Rasal1, a type of GTPase-activating protein, associates with ZAP-70 of the TCR and negatively regulates T-cell activation and tumor immunity (Thaker et al., 2019). GO annotations associated with CYB5R1 include oxidoreductase activity and cytochrome-b5 reductase activity, which act on NAD(P)H. Moreover, NADH-cytochrome b5 reductases are involved in the desaturation and elongation of fatty acids, cholesterol biosynthesis, and drug metabolism. GO annotations associated with DNAH11 include ATPase activity and microtubule motor activity.

This study also demonstrated that CIN and HRD notably increased in the PD samples, especially in the period of extramedullary plasmacytoma. Cells with genomic instability, a hallmark of cancer, have an increased tendency of underdoing genomic alterations during cell division (Negrini et al., 2010). As a result, increased genomic alterations in MM may lead to more neoantigens being expressed on the surface of PCs. Unfortunately, the immune system is naive to these novel peptides, escaping detection (Neuse et al., 2020).

Furthermore, increasing evidence in other cancers have suggested that clones with polyploid and aneuploid may be enriched at recurrence periods and mediate therapeutic resistance (Mirzayans et al., 2018; Pienta et al., 2021). These polyploid clones always exhibit a protected cellular phenotype that is largely resilience to environmental disruption. On an evolutionary timescale, polyploid programs could provide increased fitness. Upon removal of chemotherapy stress, these polyploid clones may undergo depolyploidization and generate resistant progeny. These theories may provide explanations for the results that CIN significantly increased in the PD samples in this study.

Despite the clinical success of the proteasome inhibitor bortezomib and immunomodulatory imide drugs (IMiDs), treating RRMM remains a challenge. According to the results of the WES evolutionary tree in this study, from the level of single-cell transcriptomes, malignant PCs of P1 in the final state (PD9) were further divided into three subgroups, ultimately suggesting the potential importance of the RUNX3 gene. According to literature, RUNX3 belongs to the family of RUNXs (RUNX1, RUNX2, RUNX3). In 2019, Zhou et al. (2019) demonstrated that in myeloma cells, RUNX3 and RUNX1 could interact with Ikaros family zinc finger protein 1 and 3 (IKZF1 and IKZF3). Aa a result, myeloma cell lines and primary tumors may become refractory to CRBN-dependent ubiquitylation and degradation induced by IMiDs. Moreover, inhibition of RUNX proteins resulted in enhanced sensitivity of MM cells to IMiDs. These results may improve the understanding of the complex pathophysiology of MM and provide an alternative approach for applications in personalized medicine.

This study has certain limitations. First, since the total quantity of each BM sample was stored at −80°C for a relatively long time, it was insufficient for integrated omics data, such as epigenomics and proteomics, resulting in a lack of combing data. Second, all temporal consecutive samples were obtained from only one RRMM patient, which may introduce potential bias in the interpretation of the results. Further studies with increased sample sizes could provide more definitive evidence. Third, adaptive therapy and recycle therapy require further validation in large-scale preclinical and clinical studies. In summary, this study demonstrated that temporal consecutive samples from one RRMM patient can assess the diversity of sub-clones as well as the clonal evolution trajectory driven by multi-line therapies. Moreover, distribution of driver mutations and passenger mutations were observed to be scattered in the trunk and non-trunk of the branching-pattern phylogenetic tree. Personal high-throughput sequencing that reveals specific genetic compositions and molecular phenotypes may contribute to more robust personal biomarkers and serve as guidance for personalized therapy. Furthermore, adaptive and recycle therapy that are grounded in evolutionary law may prevent a rise in the resistant PCs population and ser as a potential strategy for RRMM treatment.

## Data Availability

The clinical data of this study are restricted by the ethics committee in Zhongshan Hospital in order to protect patients' privacy. Requests to access these datasets should be directed to (Peng Liu, liu.peng@zs-hospital.sh.cn). The WES datasets used and/or analyzed during the current study are available from PRJNA690173 in SRA (https://www.ncbi.nlm.nih.gov/sra/PRJNA690173). The scRNA-seq are available from GSE188632 in GEO (https://www.ncbi.nlm.nih.gov/geo/info/linking.html).
