# Supplementary material for: Single-Cell Transcriptomes Combining with Consecutive Genomics Reveal Clonal Evolution and Gene Regulatory Networks in Relapsed and Refractory Multiple Myeloma
Source: Front Cell Dev Biol. 2022 Jan 5;9:794144. doi: 10.3389/fcell.2021.794144 (PMC8766805; doi:10.3389/fcell.2021.794144)
Supplement: Supplementary file 1 [file DataSheet1.PDF]

Supplementary Figures and Tables

1. Supplementary Figures

1.1 Supplementary Fig. 1

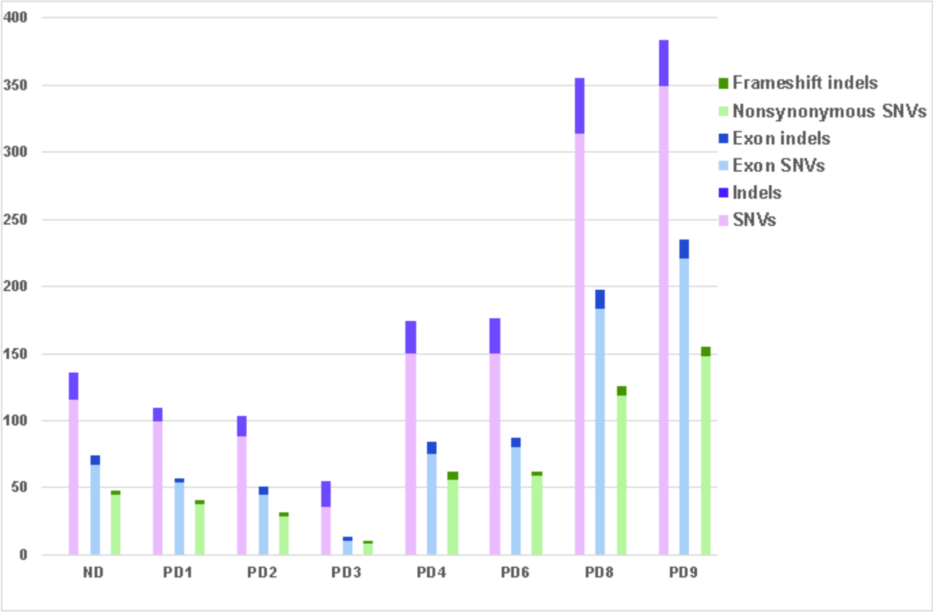

1.2 Supplementary Fig. 2

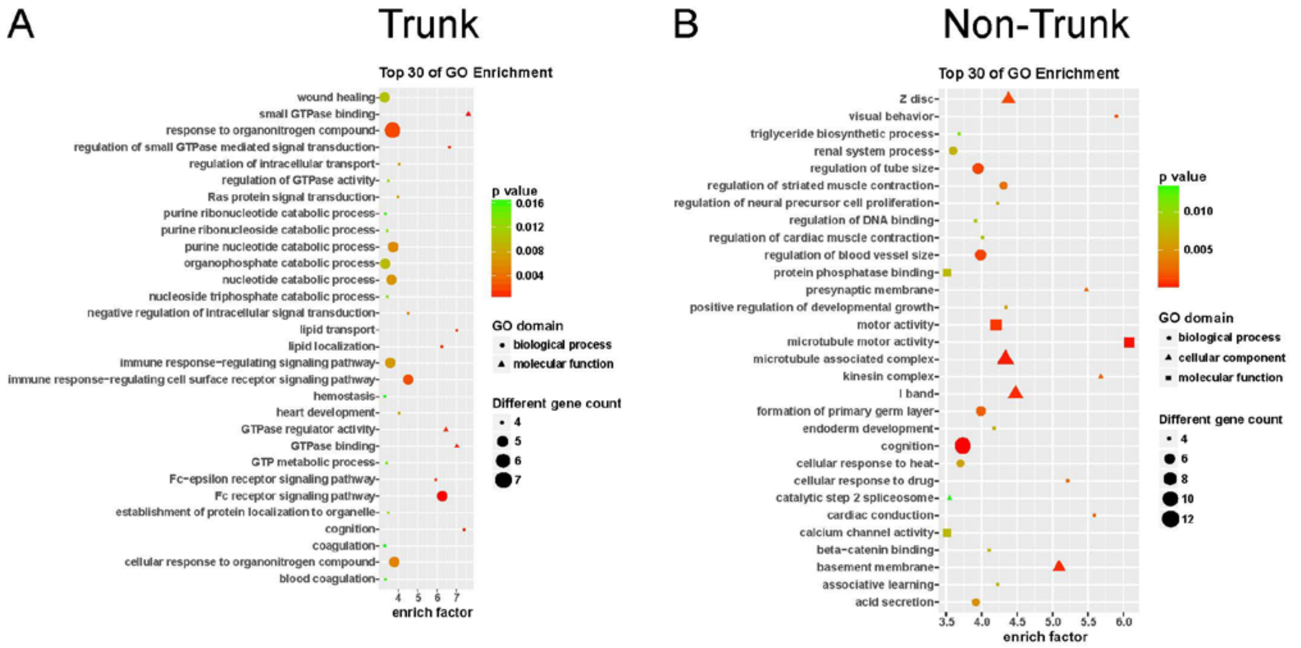

Supplementary Figure 1. In view of the heterogeneity and complex regulating network in MM progression, trunk and non-trunk genes were enriched into multiple pathways and functions using the Gene Ontology (GO) databases.

1.2 Supplementary Fig. 2

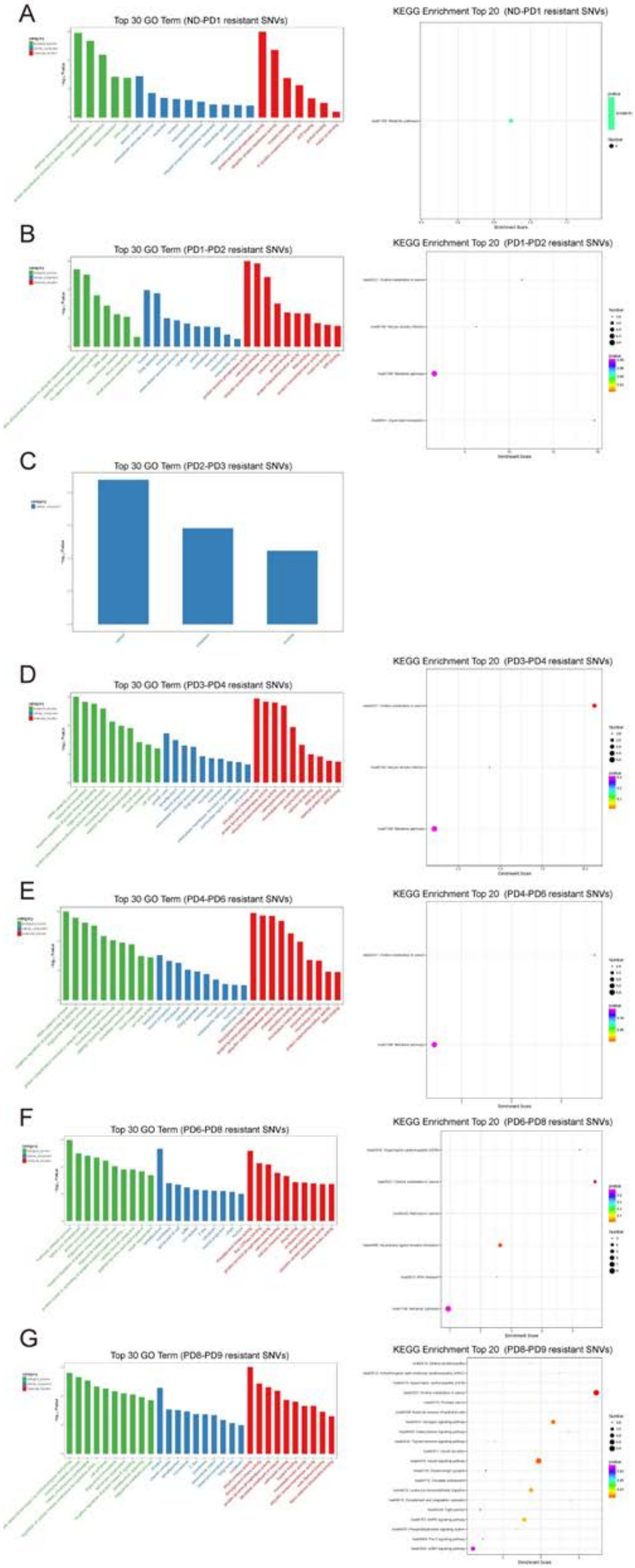

Supplementary Figure 2. Mutations of P1 were further classified into susceptible SNVs for each treatment course. GO and KEGG enrichment analyses were conducted.

1.3 Supplementary Fig. 3

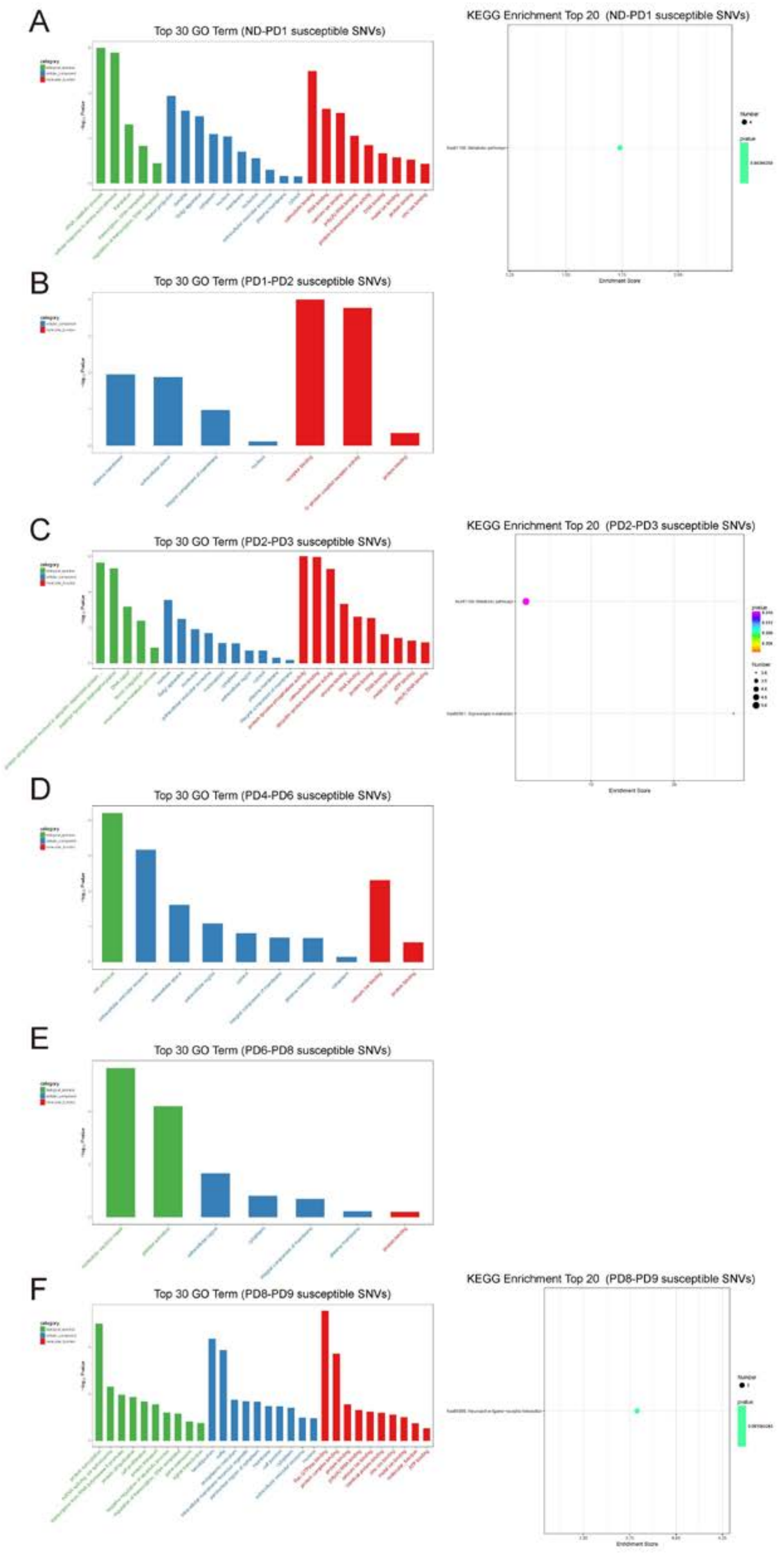

Supplementary Figure 3. Mutations of P1 were further classified into resistant SNVs for each treatment course. GO and KEGG enrichment analyses were conducted.

Supplement Table 1. Clinical characteristics of P1 patient in each progression period.

	ND	PD1	PD2	PD3	PD4	PD5	PD6	PD7	PD8 (EMP)	PD9
**Date**	2017. 7.12	2017. 12.6	2018. 4.3	2018. 8.29	2018. 11.26	2019. 4.12	2019. 6.26	2019. 10.10	2020. 3.31	2020. 8.8
**Best Response**	PR	PR	PR	SD	SD	SD	MR	PR	SD	--
**FISH (BM)**										
**del (17p)**	Negative	NA	NA	NA	NA	--	Negative	Negative	Negative	--
**del (13q14)**	+ (CN=1)	NA	NA	NA	NA	--	+ (CN=1)	+ (CN=1)	Negative	--
**1q21 gain**	+ (CN=4)	NA	NA	NA	NA	--	+ (CN=4)	+ (CN=4)	Negative	--
**t (11;14) (CCND1)**	Negative	NA	NA	NA	NA	--	Negative	Negative	Negative	--
**t (4;14) (FGFR3)**	Negative	NA	NA	NA	NA	--	Negative	Negative	Negative	--
**t (14;16) (MAF)**	Negative	NA	NA	NA	NA	--	Negative	Negative	Negative	--
**Karyotypes**	46, XX	NA	NA	NA	NA	--	46, XX	46, XX	/	--
**M protein (g/L)**	36.2	19.3	18.5	5.9	11.5	12.9	20.8	31.5	3.8	--
**HGB (g/L) (115-150)**	81	99	110	107	114	99	76	96	126	48
**WBC ($\times 10^9$/L) (3.50-9.50)**	3.98	Normal	Normal	2.4	2.60	3.82	2.91	4.47	3.10	2.28
**PLT ($\times 10^9$/L) (123-350)**	208	Normal	242	167	104	109	74	151	217	23

**Scr (μmol/L) (44-115)**	59	48	55	53	55	50	58	67	60	30
**eGFR (mL/min/1.73 m^2)**	94	101	96	97	96	98	93	83	92	115
**Ca^{2+} (mmol/L)**	Normal	2.5	2.36	2.19	2.23	2.27	2.09	2.44	2.43	2.09
**β2M (mg/L) (0.80-2.20)**	3.16	2.87	2.23	1.86	2.54	2.42	2.89	3.60	3.25	4.37
**LDH (U/L) (109-245)**	143	181	155	179	160	190	212	169	148	1555
**FLC k/λ (0.310-1.560)**	74.745	49.272	14.160	--	29.150	68.919	80.169	149.362	15.443	--
**PCs %**	35 (BM)	35 (BM)	40 (BM)	25 (BM)	20 (BM)	--	40 (BM)	50 (BM)	Negative	10 (PB)
**ECOG**	1	1	1	1	1	1	1	1	1	3

Abbreviations: ND: Newly Diagnosis; PD: Progression Disease; EMP: Extramedullary Plasmacytoma; WBC: White Blood Cells; HGB: Hemoglobin; PLT: Platelets; Scr: Serum creatinine; LDH: Lactate Dehydrogenase; FLC: Free Light Chains; PCs: Plasma Cells; BM: Bone Marrow; PB: Peripheral Blood; ECOG: Eastern Cooperative Oncology Group Performance Status; NA: Not Available.

Supplement Table 2. Summary of quality control

Samples	Raw Reads	Clean Reads	Clean Reads Percent	Raw Base	Clean Base	Clean Base Percent	GC Content	>Q20	>Q30
C	135463124	128894442	95.15%	20319468600(20.32G)	19174458754(19.17G)	94.36%	53.03%	96.73%	91.87%
ND	125669268	120004956	95.49%	18850390200(18.85G)	17882341668(17.88G)	94.86%	53.26%	96.72%	91.79%
PD1	145545460	142927472	98.20%	21831819000(21.83G)	21222916598(21.22G)	97.21%	50.45%	97.92%	94.34%
PD2	152958922	150434934	98.35%	22943838300(22.94G)	22354989172(22.35G)	97.43%	51.27%	97.94%	94.33%
PD3	128071178	125893626	98.30%	19210676700(19.21G)	18681961184(18.68G)	97.25%	51.32%	97.90%	94.25%
PD4	98474664	96737502	98.24%	14771199600(14.77G)	14357139730(14.36G)	97.20%	51.15%	97.91%	94.28%
PD6	137600052	134299380	97.60%	20640007800(20.64G)	19896308674(19.90G)	96.40%	51.32%	97.87%	94.45%
PD8	133839380	132006786	98.63%	20075907000(20.08G)	19478917994(19.48G)	97.03%	50.37%	97.35%	92.72%
PD9	113159918	111274366	98.33%	16973987700(16.97G)	16547142765(16.55G)	97.49%	51.40%	97.19%	92.36%

Supplement Table 3. Summary of mapping results

Sample	Total reads	Dup reads	Dup percent	RmDup reads	Mapped reads	Mapped Rate	On target rate	On target mean coverage	On target mean mapping quality	On target >1X coverage	On target >10 X coverage	On target >50 X coverage
Control	128894442	32848630	25.48%	96045812	95833219	99.78%	76.94%	144.7347X	57.9076	99.70%	99.30%	83.85%
ND	120004956	28753927	23.96%	91251029	91178237	99.92%	77.09%	138.3955X	57.9081	99.70%	99.20%	80.86%
PD1	142927472	16156788	11.30%	126770684	126584576	99.85%	80.47%	202.9726X	57.9203	99.66%	99.45%	96.04%
PD2	150434934	15867012	10.55%	134567922	134511380	99.96%	81.69%	219.9991X	57.9209	99.66%	99.46%	96.51%
PD3	125893626	11094879	8.81%	114798747	114759764	99.97%	81.93%	187.8043X	57.9287	99.66%	99.42%	95.17%
PD4	96737502	19690784	20.35%	77046718	77018923	99.96%	81.65%	125.6235X	57.9281	99.65%	99.28%	87.82%
PD6	134299380	21217251	15.80%	113082129	113044876	99.97%	79.57%	177.4896X	57.9006	99.69%	99.35%	90.78%
PD8	132006786	32358434	24.51%	99648352	99524339	99.88%	81.51%	161.2303X	57.8612	99.67%	99.31%	91.66%
PD9	111274366	20988141	18.86%	90286225	90190036	99.89%	80.80%	144.8003X	57.9053	99.67%	99.27%	89.82%

Supplement Table 4. Driver genes

	Chromosome	Position	Ref	Alt	P1_ND	P1_PD1	P1_PD2	P1_PD3	P1_PD4	P1_PD6	P1_PD8	P1_PD9	Func.refGene	Gene.refGene	ExonicFunc.refGene
**Non-trunk**	10	93999691	G	T	0/1 | 159,9	./. | 216,0	./. | 244,0	./. | 192,0	./. | 159,0	./. | 238,0	./. | 221,0	./. | 152,0	exonic	CPEB3	nonsynonymous SNV
**Non-trunk**	12	70965626	A	T	./. | 30,0	0/1 | 239,9	./. | 306,0	./. | 271,0	./. | 143,0	./. | 84,0	./. | 252,0	./. | 237,0	exonic	PTPRB	nonsynonymous SNV
**Non-trunk**	13	41239865	T	C	./. | 118,0	./. | 184,0	./. | 208,0	./. | 194,0	./. | 106,0	./. | 189,0	./. | 167,0	0/1 | 36,82	exonic	FOXO1	nonsynonymous SNV
**Non-trunk**	17	66547263	T	G	./. | 195,0	./. | 309,0	./. | 270,0	./. | 264,0	./. | 169,0	./. | 346,0	./. | 178,0	0/1 | 109,10	exonic	PRKAR1A	stoploss
**Non-trunk**	18	22804904	C	G	./. | 128,0	./. | 278,0	./. | 262,0	./. | 213,0	./. | 101,0	./. | 174,0	./. | 99,0	0/1 | 74,25	exonic	ZNF521	nonsynonymous SNV
**Non-trunk**	19	45855603	G	A	./. | 176,0	./. | 146,0	./. | 232,0	./. | 178,0	./. | 118,0	0/1 | 126,4	./. | 107,0	./. | 122,0	exonic	ERCC2	nonsynonymous SNV
**Non-trunk**	2	25972617	G	A	0/1 | 136,7	./. | 176,0	./. | 212,0	./. | 195,0	./. | 106,0	./. | 224,0	./. | 130,0	./. | 147,0	exonic	ASXL2	nonsynonymous SNV
**Non-trunk**	2	45233332	T	G	./. | 458,0	./. | 311,0	./. | 374,0	./. | 311,0	./. | 262,0	./. | 426,0	0/1 | 245,16	./. | 320,0	exonic	SIX2	nonsynonymous SNV
**Non-trunk**	22	29695841	A	AAGGCGAGCACC	./. | 59,0	./. | 70,0	./. | 91,0	./. | 82,0	0|1 | 40,4	./. | 52,0	./. | 65,0	./. | 57,0	exonic	EWSR1	frameshift insertion
**Non-trunk**	22	29695843	T	TCAGGAGCGCAGAGATCGGCCCTACTAGATGCAGAGACCCCGC	./. | 59,0	./. | 72,0	./. | 90,0	./. | 83,0	0|1 | 40,4	./. | 52,0	./. | 64,0	./. | 55,0	splicing	EWSR1	.
**Non-trunk**	22	41564778	TCTTTGC	T	./. | 145,0	./. | 232,0	./. | 303,0	./. | 245,0	./. | 145,0	./. | 228,0	./. | 169,0	0/1 | 143,10	exonic	EP300	nonframeshift deletion
**Non-trunk**	7	86416173	G	A	0/1 | 381,10	0/1 | 586,6	./. | 739,0	./. | 587,0	./. | 456,0	./. | 524,0	./. | 542,0	./. | 688,0	exonic	GRM3	stopgain
**Non-trunk**	7	138596009	C	T	./. | 49,0	./. | 45,0	./. | 69,0	./. | 52,0	./. | 43,0	0/1 | 65,3	./. | 102,0	./. | 79,0	exonic	KIAA1549	nonsynonymous SNV
**Non-trunk**	8	114031337	T	C	./. | 121,0	./. | 321,0	./. | 263,0	./. | 232,0	./. | 138,0	./. | 210,0	./. | 238,0	0/1 | 107,5	exonic	CSMD3	nonsynonymous SNV
**Non-trunk**	9	96051868	C	G	./. | 196,0	./. | 148,0	./. | 168,0	./. | 160,0	./. | 102,0	./. | 206,0	0/1 | 37,20	0/1 | 74,23	exonic	WNK2	nonsynonymous SNV
**Trunk**	12	112888165	G	T	./. | 67,0	0/1 | 98,4	0/1 | 110,8	./. | 144,0	0/1 | 51,9	0/1 | 72,15	0/1 | 74,58	0/1 | 50,43	exonic	PTPN11	nonsynonymous SNV
**Trunk**	16	2120479	C	T	0/1 | 248,10	./. | 358,0	0/1 | 277,7	0/1 | 258,6	0/1 | 121,27	0/1 | 143,27	0/1 | 63,51	0/1 | 257,58	exonic	TSC2	nonsynonymous SNV
**Trunk**	2	60688215	T	G	0/1 | 267,45	./. | 357,0	./. | 370,0	./. | 340,0	./. | 269,0	0/1 | 264,45	0/1 | 189,67	0/1 | 167,65	exonic	BCL11A	nonsynonymous SNV
